# Factors influencing the long-term prognosis of root tip resected teeth

**DOI:** 10.3205/iprs000139

**Published:** 2019-09-02

**Authors:** Andreas Sakkas, Karsten Winter, Maximilian Rath, Frank Mascha, Sebastian Pietzka, Alexander Schramm, Frank Wilde

**Affiliations:** 1 Department of Oral and Plastic Maxillofacial Surgery, Armed Forces Hospital Ulm, Germany; 2 Institute of Anatomy, Medical Faculty, University of Leipzig, Germany; 3 Department of Oral and Maxillofacial Surgery, University Hospital Ulm, Germany

**Keywords:** apical surgery, healing, prognostic factors, endodontics

## Abstract

**Introduction: **The aim of the study was to investigate possible predictive factors influencing the long-term success of root tip resection.

**Methods:** The retrospective study included 216 patients (♂ 111, ♀ 106, median age 43.3 years). A total of 261 root tip resections were performed on these patients between 1989 and 2012. In addition to determining the success rates 5 and 10 years postoperatively, the factors gender, age, tooth type, use of bone replacement material and preoperative periodontal tooth status were examined with regard to their significance for the long-term prognosis of root tip resected teeth.

**Results:** The evaluation showed an average success rate of 63.6% for all included teeth over the entire observation period (tooth at least one year postoperatively still in situ). The 5-year success rate was 78.2%, the 10-year success rate 63.1%. A dependence of the success rates on the tooth type could not be evaluated. However, the examination showed a clear dependence of the success on the age of the patients. Root tip resections in patients in the age group 60 years and older had significantly worse success rates compared to the age groups 20 to 39 years and 40 to 59 years. The prognosis was also significantly better for patients in the age group 20 to 39 years than for patients in the age group 40 to 59 years. Periodontally compromised teeth showed only a tendency for a poorer prognosis than periodontally healthy teeth. With regard to sex and intraoperative filling of the resection defect with bone replacement material, no differences in the success rates were found.

**Conclusions:** A root tip resection is a good option, largely independent of the type of tooth, to preserve a tooth in the medium to long term after unsuccessful endodontic treatment. However, a revision of the endodontic treatment or even an extraction with subsequent implantation should always be considered as an alternative, especially with increasing age.

## Zusammenfassung

**Hintergrund:** Ziel der Studie war die Untersuchung möglicher prädiktiver Einflussfaktoren auf den Langzeiterfolg der Wurzelspitzenresektion.

**Methode:** In die retrospektive Studie konnten 216 Patienten (♂ 111, ♀ 106, medianes Alter 43,3 Jahre) eingeschlossen werden. Bei diesen waren im Zeitraum 1989 bis 2012 insgesamt 261 Wurzelspitzenresektionen durchgeführt worden. Neben der Ermittlung der Erfolgsraten 5 und 10 Jahre postoperativ wurden die Faktoren Geschlecht, Alter, Zahngattung, Einsatz von Knochenersatzmaterial sowie der präoperative parodontale Zahnstatus hinsichtlich der Bedeutung für die Langzeitprognose wurzelspitzenresezierter Zähne untersucht.

**Ergebnisse:** Die Auswertung ergab bezogen auf alle eingeschlossenen Zähne, betrachtet über den gesamten Beobachtungszeitraum, eine mittlere Erfolgsquote (Zahn mindestens 1 Jahr postoperativ noch in situ) von 63,6%. Die 5-Jahres Erfolgsrate lag bei 78,2%, die 10-Jahres Erfolgsrate bei 63,1%. Eine Abhängigkeit der Erfolgsraten von der Zahngattung (Ober-/Unterkiefer-Frontzähne, Ober-/Unterkiefer-Prämolaren, Ober-/Unterkiefer-Molaren) konnte nicht evaluiert werden. Die Untersuchung zeigte jedoch eine eindeutige Abhängigkeit des Erfolgs vom Alter der Patienten. Dabei hatten Wurzelspitzenresektionen bei Patienten in der Altersgruppe 60 Jahre und älter im Vergleich zu den Altersgruppen 20 bis 39 Jahre und 40 bis 59 Jahre signifikant schlechtere Erfolgsraten. Auch war die Prognose bei Patienten der Altersgruppe 20 bis 39 Jahre signifikant besser als bei Patienten in der Altersgruppe 40 bis 59 Jahre. Parodontal geschädigte Zähne zeigten lediglich eine Tendenz für eine schlechtere Prognose im Vergleich zu parodontal gesunden Zähne. Bezüglich des Geschlechts sowie einer intraoperativen Auffüllung des Resektionsdefektes mittels Knochenersatzmaterial wurden keine Unterschiede in Bezug auf die zu erwartenden Erfolgsraten sichtbar.

**Schlussfolgerung:** Eine Wurzelspitzenresektion stellt eine gute, von der Zahngattung weitestgehend unabhängige Option dar, um einen Zahn nach einer nicht erfolgreichen endodontischen Behandlung mittel- bis langfristig zu erhalten. Eine Revision der endodontischen Behandlung oder eine Extraktion ggf. mit anschließender Implantation sollten jedoch immer, insbesondere im höheren Alter, als Alternative in Betracht gezogen werden.

## Introduction

Root tip resection is a common dental-surgical therapy option. It describes the surgical shortening of the root apex by approx. 3–4 mm and the removal of the pathological surrounding tissue after the creation of a “bone window” by means of osteotomy. The operation is performed either in combination with preoperative or intraoperative orthograde root canal filling, which can optionally be performed retrograde as well. A root tip resection is used for teeth with persistent acute apical or chronic apical periodontitis and as a primary option for teeth with very strong root curvature or after unsuccessful endodontic revision treatment. As a last consequence, it must be regarded as the last tooth preserving treatment option. The main indication of root tip resection is chronic apical periodontitis. Epidemiological studies in western industrial countries have shown that 1.5–7% of all teeth suffer from chronic apical periodontitis [1], [2], [3]. Thus, it becomes clear that the root tip resection will continue to have its significance in the future. 

While information is available on prognostic factors for root tip resection, most clinical studies evaluate the outcome in terms of the root filling material used in endodontic treatment. Few studies evaluate possible prognostic factors, such as patient age, sex, tooth type, or intraoperative filling of the resection defect with bone grafting material. Studies evaluating several prognostic factors simultaneously in relation to the healing results of apical surgery are also rare [4], [5], [6].

Due to the importance of root-tip resection in dentistry, it seems relevant to provide patients with profound information on the expected success of root-tip resection.

The aim of this study was therefore to identify patient-dependent and surgical parameters that could be relevant as possible predictive factors for long-term success after root tip resection.

## Methods

Patient recruitment and data collection for this study took place in a high standard German private dental practice under the supervision of the Department of Maxillofacial and Facial Plastic Surgery, Armed Forces Hospital Ulm, Germany. The research was conducted in full accordance with ethical principles, including the World Medical Association Declaration of Helsinki. Data were anonymized and de-identified prior to analysis. Reporting was based on the recommendations of the Strengthening the Reporting of Observational Studies in Epidemiology (STROBE) initiative [7]. The study was approved by the Ethics Commission of the University of Ulm on 14.06.2017.

The data collected from the medical records were documented using the Excel Mac 2011^©^ software. The medical records of all patients included were completely available, so that the entire dental history could be reconstructed. 

The data was based on the retrospective analysis of 216 patient cases in which root tip resection had been performed on at least one permanent tooth over a period of 23 years (October 1989 to September 2012 inclusive). A total of 261 root-tip resections were performed on these 216 patients. The inequality between the number of patients (n=261) and the number of root-tip resections performed (n=261) is due to the fact that several root-tip resections were performed on some patients during the observation period. 105 of these patients were female and 111 were male. The average age at the time of surgery was 43.1 years, the youngest patient was 14, the oldest 75 years old. 

### Inclusion criteria

To be included in the study, the following parameters had to be documented in detail in the patient file:

- Affected tooth
- Indication for root tip resection
- Preoperative periodontal dental status
- Use of bone replacement material for defect filling (Yes/No)
- Primary surgery or revision surgery
- At least one documented follow-up appointment
- Presence of a complete radiographic history, both directly postoperatively and over the course of time

### Exclusion criteria

Excluded from the study were patients whose file cards did not document one or more of the aforementioned criteria, and patients who had not been recalled for ten years or more. Due to the lack of numbers, no root tip resections with retrograde root fillings were included in the study.

### Analysis criteria

For data analysis, a catalogue of criteria was created in which the findings documented in the patient file were transferred. 

The following criteria were collected:

- Gender
- Age at the time of root tip resection.
  For further evaluation, the patients were further subdivided into the following age groups:
  - <20 years
  - 20–<40 years
  - 40–<60 years
  - ≥60 years
- Tooth type: maxillary frontal teeth, maxillary premolars, maxillary molars, mandibular frontal teeth, mandibular premolars, mandibular molars
- Use of bone substitute material for apical defect filling after root tip resection
- Preoperative periodontal condition: periodontally healthy versus periodontally compromised teeth. This condition was checked via records in the files and x-rays. If the preoperative clinical and radiological findings contained no evidence of periodontal impairment, the tooth was classified as periodontally healthy. If there was clinical or radiological evidence of a vertical or horizontal bone defect, a furcation involvement of grade II/III or a degree of tooth loosening of grade II/III, the tooth was classified as preoperatively periodontally compromised.
- Indication for root tip resection.
  A distinction was made between the following indications:
  - Abscess
  - Apical periodontitis
  - Apical root fracture
  - Chronic apical periodontitis (fistula duct)
  - Root canal instrument fracture
  - Root canal obliteration
  - Apical root third tooth perforation
  - Overpressed root filling material
  - Primary root tip resection versus revision root tip resection

### Success criteria

The so-called operation success in months was determined on the basis of the operation date and the last documented check-up date.

The root tip resection was considered a success if the following findings were present:

- Affected tooth continued in situ for at least twelve months after root tip resection
- Tooth on percussion negative
- No bite problems at all
- No apical pain
- X-ray ossification visible
- No complaints subjectively perceived by the patient

Root tip resection was considered a failure if the following findings were present:

- Extraction of the root tip resected tooth
- Persistent apical periodontitis
- Chronic apical periodontitis with fistula tract
- Pain on apical pressure
- No periapical ossification visible on radiography

In addition, a minimum of twelve months of root tip resection was required for inclusion. In contrast, failures were included without a minimum requirement.

### Inclusion and exclusion criteria for the 5-year and 10-year success rates

The 5- and 10-year success rates were determined on the basis of the success of the operation in months. The following special inclusion and exclusion criteria were defined.

The 5-year success rate included all teeth evaluated as successful with a success duration of at least 60 months (n=178). All teeth that were resected less than 60 months before the date of data collection could not be included in the 5-year success rate (n=83).

The 10-year success rate included all resected teeth with a success duration of at least 120 months (n=110). All teeth operated on less than 120 months before the date of data collection were not included in the 10-year success rate (n=151). 

### Operative procedure

The root tip resections were performed as follows. The procedure was performed using 2.5x magnifying glasses. After local anesthesia a gingival incision was made. The extent of the incision was individually adjusted to achieve an ideal overview. The bony preparation was carried out using burs of different sizes under constant rinsing with a 0.9% natrium chloride solution. The bony window to the root tip was designed in such a way that the affected root tip was completely visible. The affected root apex was shortened with a Lindemann cutter, whereby a slight bevel to the vestibular side was used for a better examination. The periapical granulation tissue was removed using a curette. If a cyst was present, it was excised. The resected root was then thoroughly inspected. In particular, the apical quality of the preoperatively performed orthograde root filling and the presence of cracks in the root were checked visually and tactilely with the aid of a probe. If the root filling proved to be tight, the resected surface was smoothed and the cavity was extensively rinsed with saline solution. If the root filling was found to be insufficient, the root stump was further shortened. If the root canal filling was again considered insufficient after this resection, an intraoperative orthogonal revision of the root canal filling was performed. The root canal was prepared orthograd manually or mechanically, disinfected by rinsing with NaOCl (3%) and H_2_O_2_ (3%). The root canal was filled with gutta-percha pins as well as sealers (Endomethasone N septodent^®^ or AH26 Dentsply^®^) employing lateral and vertical condensation. The gutta-percha pin protruding in the resection site was removed carefully. Subsequently, the root stump was smoothed and cleaned and the apical sealing of the root filling was checked again. Depending on the size of the bone defect, it was decided in each individual case to use bone replacement material to fill the defect. However, the decision was not subject to any clearly objective criteria. When using bone replacement material, preparations such as FRIOS Algipore (Dentsply^®^), Bio Oss (Geistlich^®^) or Bone Ceram (Straumann^®^) were used. In cases of inflammation-related or iatrogenic damage to the periosteum, the bony defect was additionally covered with a resorbable bio-membrane (Bio-Gide Geistlich^®^) prior to suture closure. Finally, primary wound closure was achieved with single button sutures using a synthetic, monofilament, non-absorbable suture material (Ethilon Polyamide 6 3-0, Ethicon^®^). 

Postoperatively, directly after the operation, an X-ray control was performed. A dental X-ray film of the affected tooth was made for this purpose. If the radiological findings revealed a need for correction, this was carried out immediately. If there was no wound healing disorder or other special feature, the sutures were removed after seven days postoperatively. 

### Statistical evaluation

The collected data and results were collected using Excel Mac 2011^©^ and then statistically evaluated by IBM SPSS Statistics^©^. Chi-square test was used to check the statistical significance of the interdependencies between the individual groups. A p-value of ≤0.05 was defined as significant, a p-value of ≤0.005 as highly significant. 

Furthermore, the success rates were visualized by Kaplan-Meier curves using IBM SPSS Statistics^©^.

## Results

Of 261 root-tip resections performed, 166 were considered successful (63.6%) and 95 were considered unsuccessful (36.4%) in the observation period. 22 teeth had a postoperative success of at most 50 months, 27 teeth a postoperative success of at least 50 and at most 100 months, 51 teeth a postoperative success of at least 150 and at most 200 months, and 49 teeth a postoperative success of at least 200 and at most 250 months.

**Figure 1:**
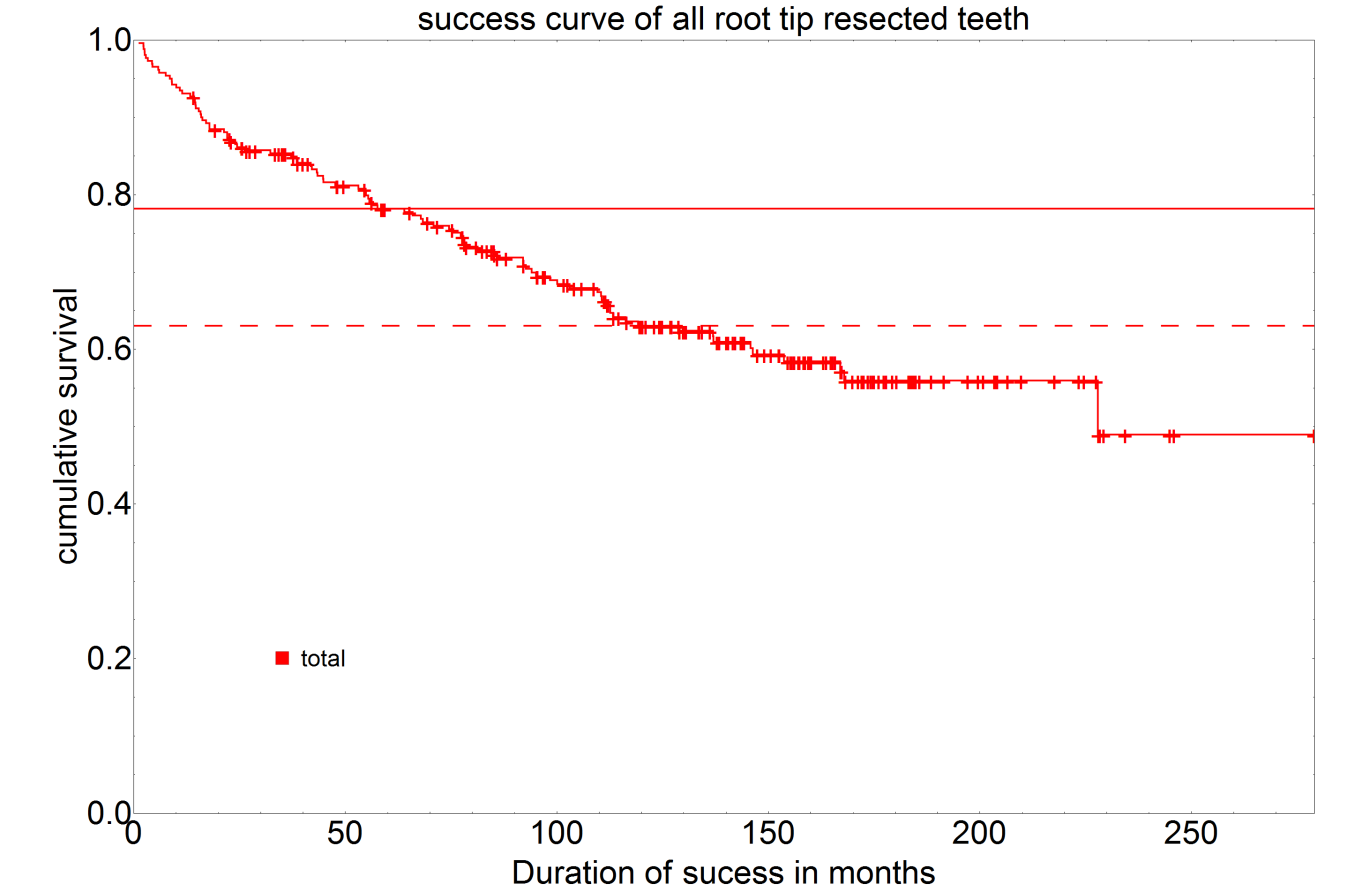
Illustration of the success curve of all root tip resected teeth (n=261) using the Kaplan-Meier survival function. The intersection of the curve with the solid horizontal line stands for the 5-year success rate, the intersection with the dashed horizontal line for the 10-year success rate.

The evaluation showed a 5-year success rate of 78.2% and a 10-year success rate of 63.1% for all included root tip resections in this study (Figure 1 (Fig. 1)).

### Success depending on gender

In this study, 126 teeth of the 261 teeth were resected in female patients and 135 teeth in male patients. In the female patients, 74 (58.7%) teeth were considered successful and 52 (41.3%) teeth were considered unsuccessful. Of the 135 teeth of male patients, 92 (68.1%) were evaluated as success and 43 (31.9%) as failure. 

**Figure 2:**
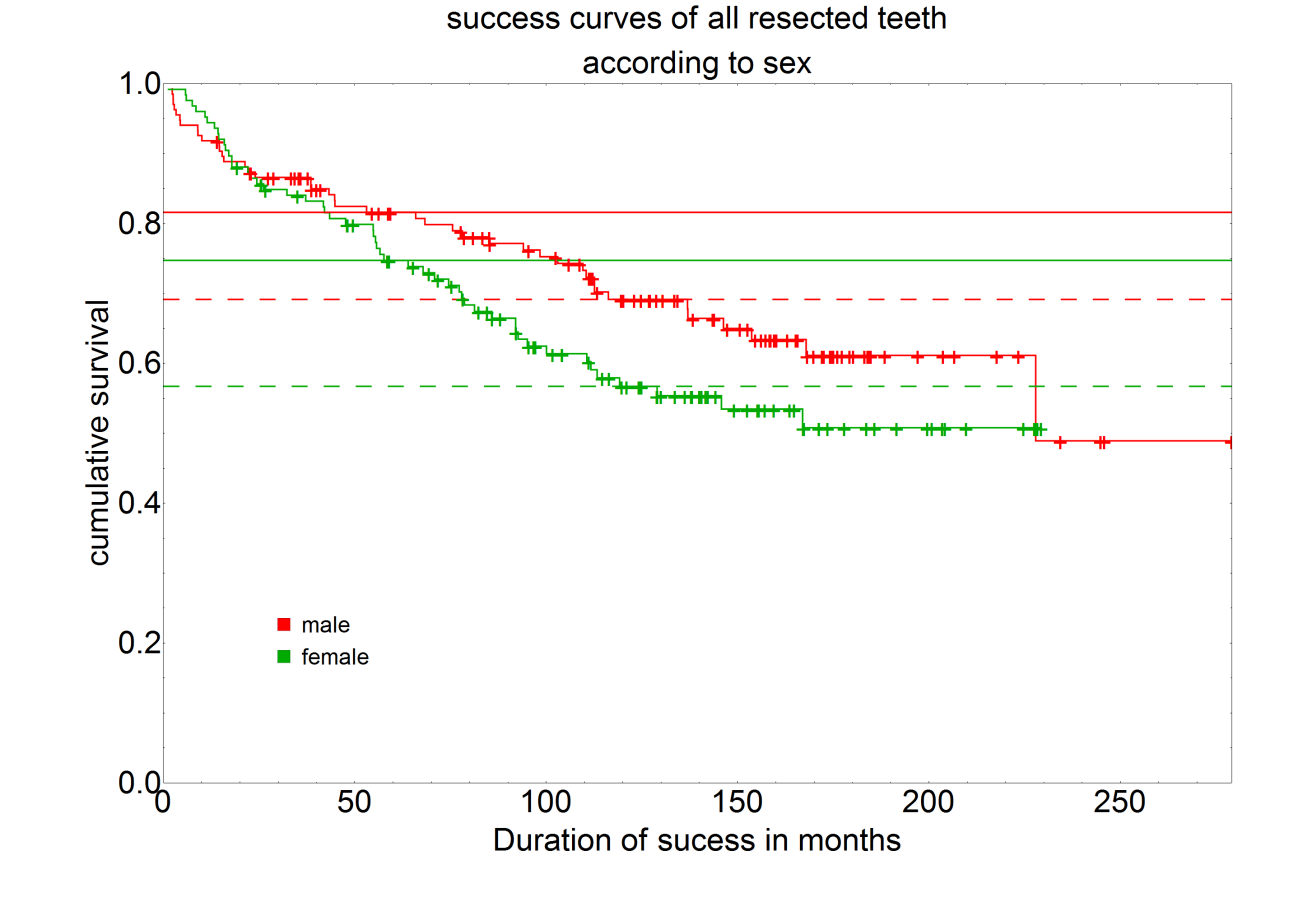
Representation of the success curves of all resected teeth, separated by sex (f=female, n=126 and m=male, n=135) using the Kaplan-Meier survival function. The intersection of the curves with the solid horizontal line stands for the 5-year success rate, the intersection with the dashed horizontal line for the 10-year success rate.

On the basis of the success curves of all resected teeth, separated by sex, a 5-year success rate of 74.4% and a 10-year success rate of 56.7% can be seen for the teeth of female patients. For teeth of male patients, a 5-year success rate of 81.6% and a 10-year success rate of 69.1% can be seen (Figure 2 (Fig. 2)). 

A comparison of both gender-specific success rates showed no statistically significant difference (P=0.109).

### Success depending on age groups

With 82 successfully operated teeth of n=112 and a success rate of 73.2%, the age group of 20 to under 40 years of age is the group with the most successfully operated teeth. The age group over 40 to under 60 years follows with a success rate of 60.2% (62 successfully operated teeth of n=103). The under-20 age group has a success rate of 57.1% (4 successfully operated teeth of n=7). In contrast, the age group of at least sixty years of age with a success rate of 46.2% (18 successfully operated teeth of n=39) is the age group with the lowest success rate.

**Figure 3:**
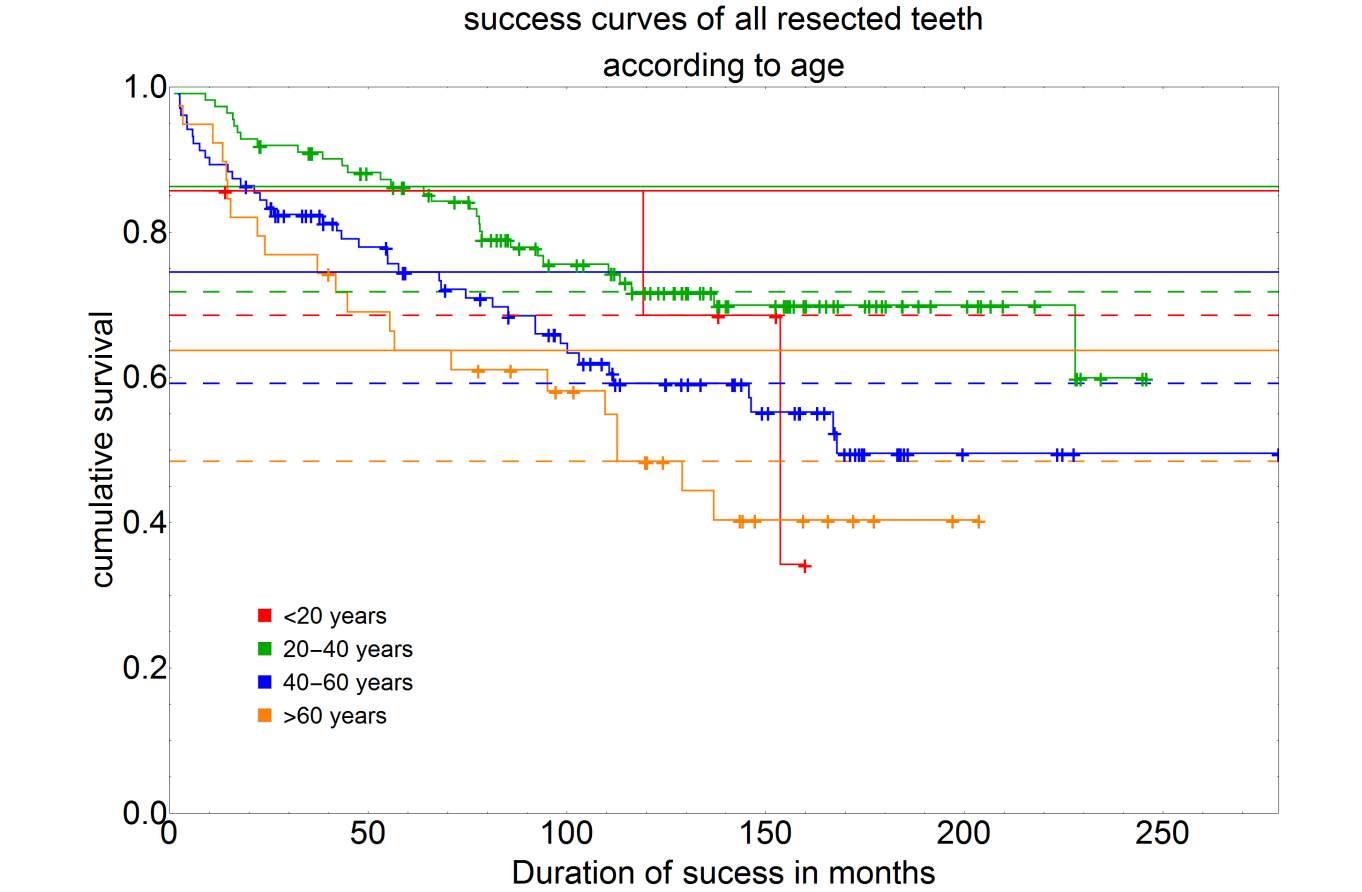
Representation of the survival curve separated by age groups (<20, n=7; 20–<40, n=112; 40–<60, n=103; ≥60, n=39) using the Kaplan-Meier survival function. The intersection of the curves with the solid horizontal line stands for the 5-year success rate, the intersection with the dashed horizontal line for the 10-year success rate.

Based on the survival curves of all resected teeth separated according to age groups, a 5-year success rate of 85.7% and a 10-year success rate of 68.6% can be identified for the group of under 20-year-olds. For the group of 20 to under 40-year-olds a 5-year success rate of 86.3% and a 10-year success rate of 71.8% can be seen. The group of 40- to under 60-year-olds has a 5-year success rate of 74.5% and a 10-year success rate of 59.2%. For those at least 60 years of age the curve shows a 5-year success rate of 63.7% and a 10-year success rate of 48.5% (Figure 3 (Fig. 3)).

A comparison of the 20 to under 40-year-old age group with the 40 to under 60-year-old age group showed a statistically significant difference with a P-value of 0.019. The comparison of the age group 20 to under 40 years with the age group at least 60 years showed a statistically highly significant difference with a P-value of 0.001. The comparison of the age group 20 to under 40 years with the age group at least 60 years even showed a statistically highly significant difference with a P-value of 0.001. This shows that the age group of 20 to under 40-year-olds has statistically significantly better success rates than the age group of 40 to under 60-year-olds or the age group of at least 60-year-olds. On the other hand, there was no statistically significant difference between the age group of 40 to under 60 years and the age group of at least 60 years. A statistical comparison with the age group under 20 years was not possible because this group was the numerically smallest of all age groups with only seven included root tip resections and therefore a comparison would not be statistically representative.

### Success depending on tooth type 

The general success rates depending on the tooth type showed a variability between 77.8% and 57.8%. With a success rate of 77.8% the maxillary molars (28 successfully operated teeth of n=36) are the teeth with the highest success rate of this study. The lower front teeth followed with a success rate of 76.9% (10 successfully operated teeth of n=13). The lower premolars showed a success rate of 75% (15 successfully operated teeth of n=20), the upper front teeth a success rate of 60.6% (43 successfully operated teeth of n=71). The lower jaw molars had a success rate of 57.9% (33 successfully operated teeth of n=57), the upper jaw premolars 57.8% (37 successfully operated teeth of n=64).

**Figure 4:**
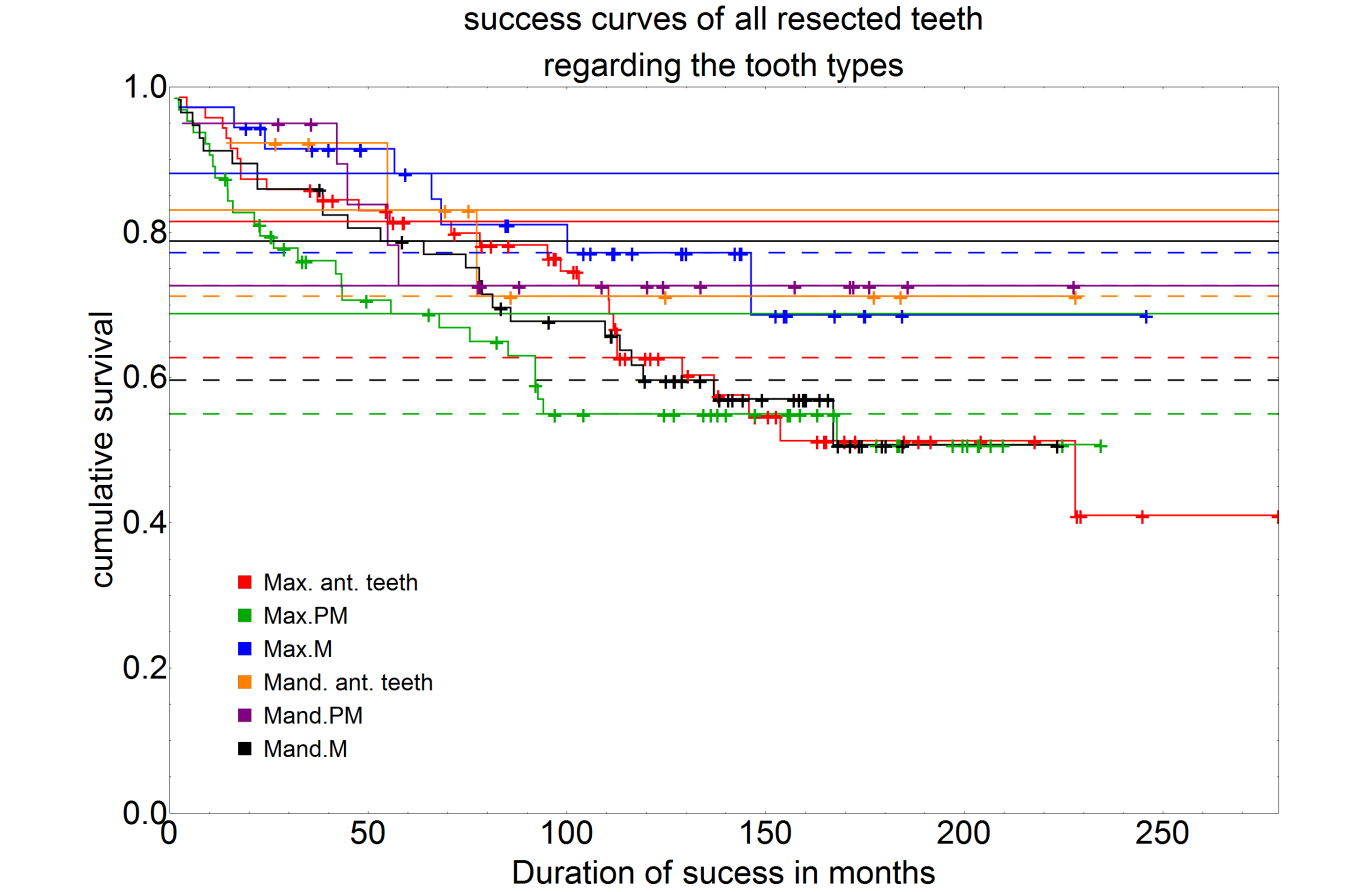
Illustration of the survival curve regarding the tooth types (Max. ant. teeth = maxillary anterior teeth, n=71; Max.PM=maxillary premolars, n=64; Max.M=maxillary molars, n=36; Mand. ant. teeth=mandibular anterior teeth, n=13; Mand.PM=mandibular premolars, n=20; Mand.M=mandibular molars, n=57) using the Kaplan-Meier survival function. The intersection of the curves with the solid horizontal line stands for the 5-year success rate, the intersection with the dashed horizontal line for the 10-year success rate.

For the maxillary anterior teeth, the 5-year success rate was 81.5% and the 10-year success rate 62.7%. The maxillary premolars showed a 5-year success rate of 68.8% and a 10-year success rate of 55.0%. The maxillary molars had a 5-year success rate of 88.1% and a 10-year success rate of 77.2%. The lower anterior teeth had a 5-year success rate of 83.1% and a 10-year success rate of 71.2%. For mandibular premolars, both the 5-year success rate and the 10-year success rate were 72.6%. A 5-year success rate of 78.8% and a 10-year success rate of 59.6% were observed for the lower jaw molars (Figure 4 (Fig. 4)).

However, a comparison of the success rates within the individual tooth types showed no statistically significant differences.

### Success prognosis depending on the use of bone graft substitutes

29 of the 45 teeth that were treated with bone replacement material during surgery were considered successful. This corresponds to a percentage of 64.4%. In 216 teeth, however, no bone replacement material was used. Of these 137 root tip resections were considered successful. This corresponds to 63.4%. 

**Figure 5:**
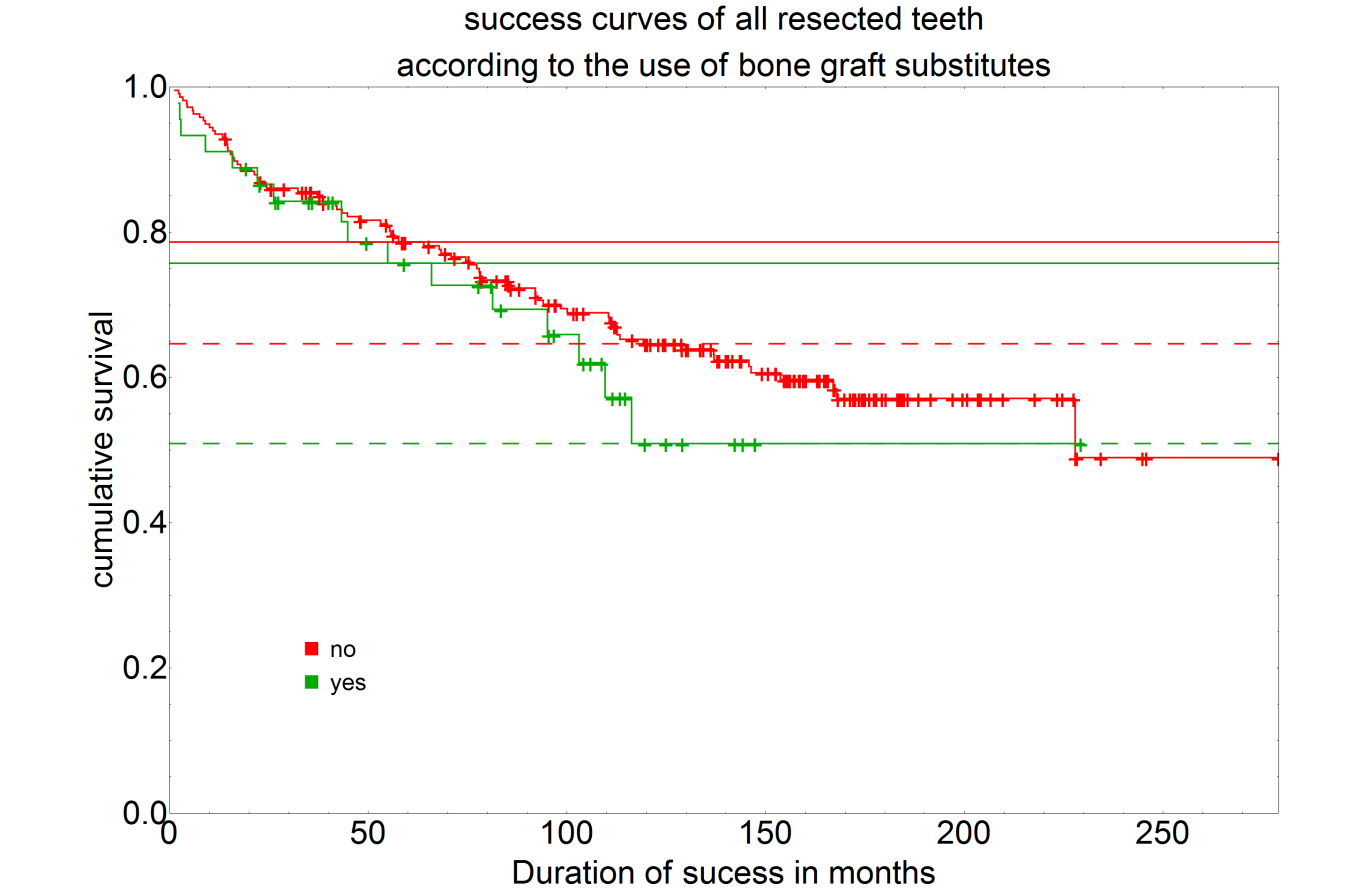
Survival curves separated by the use of bone graft substitutes (no, n=216; yes, n=45) using the Kaplan-Meier survival function. The intersection of the curves with the solid horizontal line stands for the 5-year success rate, the intersection with the dashed horizontal line for the 10-year success rate.

For the teeth for which bone replacement material was used during resection, the 5-year success rate was 75.7% and the 10-year success rate 50.1%. For teeth for which no bone replacement material was used during resection, however, a 5-year success rate of 78.6% and a 10-year success rate of 64.6% were observed (Figure 5 (Fig. 5)).

The comparison of the success rates of the groups with and without the use of bone graft substitutes showed no statistically significant difference with a P-value of 0.397, so that for the intraoperative use of bone graft substitutes no benefit for the prognosis of a root tip resection could be determined.

### Success with regard to the preoperative condition of the periodontium

Of the 202 preoperatively periodontally healthy teeth 133 were assessed as successful (65.8%). Of the 59 preoperatively periodontally compromised teeth 33 were successfully treated by root tip resection (55.9%).

**Figure 6:**
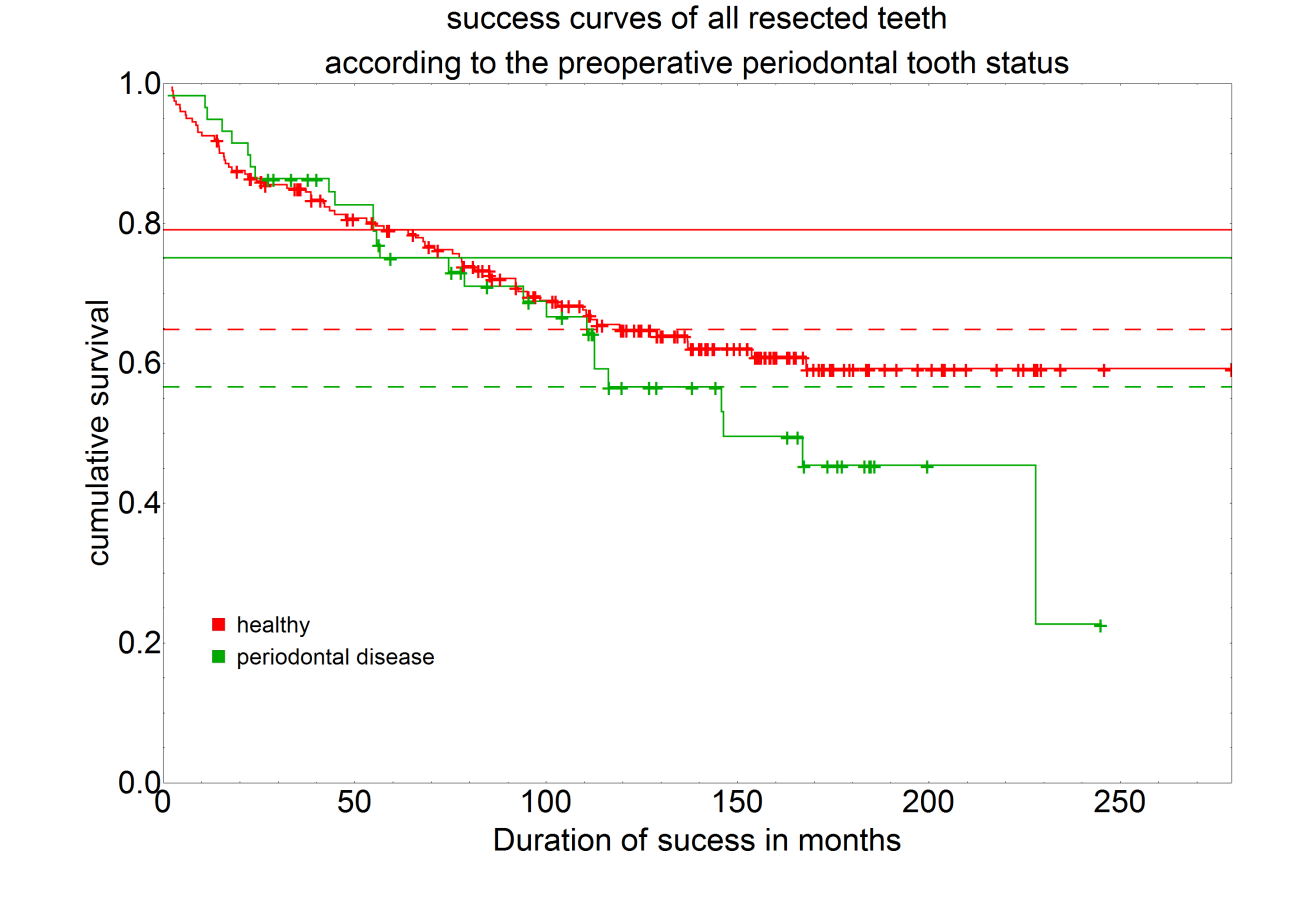
Survival curves separated by preoperative periodontal tooth status (healthy, n=216; periodontally compromised, n=59) using the Kaplan-Meier survival function. The intersection of the curves with the solid horizontal line stands for the 5-year success rate, the intersection with the dashed horizontal line for the 10-year success rate.

Teeth whose periodontium was assessed as preoperatively healthy showed a 5-year success rate of 79.1% and a 10-year success rate of 64.8%. Teeth whose periodontium was assessed as preoperatively compromised showed a 5-year success rate of 75.1% and a 10-year success rate of 56.6% (Figure 6 (Fig. 6)).

However, the statistical comparison between these two groups showed no significance (P-value=0.252).

### Root tip resection revisions

Among the n=261 included root tip resections only 16 revision operations were found. Of these, 9 were successful (56.3%). Of the 245 primary surgeries, 164 were successful, which corresponds to a rate of 66.9%. Due to the very different group sizes of the two groups to be compared (primary operation n=245 to revisions n=16) a meaningful statistical evaluation was unfortunately not possible.

### Root tip resection indications

The numerical distribution of the individual indications showed a considerable variance (abscesses n=10; apical periodontitis n=206; apical root fracture n=4; chronic apical periodontitis n=9; instrument fracture n=9; obliteration of the root canal n=4; perforation in the apical root third n=7; overfilled root filling material n=12). A meaningful statistical evaluation was therefore not possible.

## Discussion

The data from this study were analyzed with regard to the significance of various factors for the long-term survival of root tip resected teeth. The special feature was that all root tip resections included in this study were performed by one and the same surgeon. This fact makes it possible to identify factors influencing the success of root tip resection that are independent of the surgeon. In comparison to other published studies (Table 1(Tab. 1)), data from a very long observation and treatment period of almost 23 years were evaluated in this study as further special feature. On the other hand, the treatment result is naturally also influenced by the skills and experience of the surgeon. Therefore, the results of this work cannot be generally applied to every practitioner.

Studies on the prognosis after root tip resection are often difficult to compare because they usually vary in the number of included cases, the number of surgeons, the indication, the definition of success or failure and the duration of the observation period (Table 1 (Tab. 1)). 

Of the 261 resected teeth, 166 were considered a success and 95 a failure. The 5-year success rate was 78.2%, the 10-year success rate 63.1%. In comparison to the results described in the literature, these results are rather in the lower range (Table 1 (Tab. 1)). This can possibly be explained by the long observation period of this study in comparison to all other studies. In addition, strict inclusion and exclusion criteria were established in this study as well. The success and failure of a root tip resection was also clearly defined in this study. For example Gagliani et al. showed in their study a 5-year success rate of 86% for a collective of 162 root-tip resected teeth [8]. However, they excluded periodontally compromised teeth with probing depths >6 mm from their study. Although periodontal pre-damage could not be statistically filtered out in this study as a significant feature for a poorer prognosis, our study shows a clear tendency towards a poorer long-term prognosis of periodontally impaired teeth. Unfortunately, this tendency and this connection cannot be proven by further studies, since the studies available to date have not specifically dealt with this problem. Song et al. in their study (n=172) even describe a 5-year success rate of 91.5% [9]. However, the aftercare interval for 65% of her patients was not more than 2 years, so that they had less than 50% of included teeth available to determine their 5-year success rate. Furthermore, the investigated collective consisted to a large extent (n=102) of frontal teeth, which probably also influenced the very high 5-year success rate of 91.5%. Interestingly, this contradicts the results of this study, which could not filter out any dependence of success on the type of teeth, although with the exception of the lower frontal teeth, a quite comparable number of teeth could be assigned to the individual teeth types. Based on clinical experience, this was not expected by the authors beforehand, as the surgical and endodontic requirements, especially for molars and maxillary premolars, would have meant that a difference could have been expected in comparison, for example, with maxillary frontal teeth. This assumption was supported by several further studies which were able to establish such a connection in their investigations [5], [10], [11]. Nevertheless, there are also numerous studies which come to the same conclusion as the one here, namely that the success of a root tip resection in principle does not seem to depend on the type of tooth [12], [13], [14].

The 261 root-tip resections examined here are divided into 126 teeth of female and 135 teeth of male patients. In percentage terms, this corresponds to a gender-specific distribution of 48.3% female to 51.7% male. Thus, an equal gender distribution can be assumed. An overweight of female patients undergoing a root-tip resection, which was repeatedly described in the literature and explained by Jørnung and Fardal by an increased dental aesthetic awareness, could therefore not be confirmed in this study [15]. As expected, there was no dependence of success on sex, which is in line with the results of other studies [6], [12], [14], [16], [17].

The present study shows a clear dependence of the prognosis after root tip resection on the age of the patient. The statistical comparison showed that root-tip resections in patients in the age group 60 years and older have the lowest prognosis compared to the age groups 20 to 39 years and 40 to 59 years. The prognosis was also significantly better for patients in the age group 20 to 39 years than for patients in the age group 40 to 59 years. A possible explanation for this connection could be increasing comorbidities with increasing age. However, these results are not supported by other studies [5], [9], [12], [18].

The use of bone graft substitutes in apical surgery is controversially discussed in the literature. In their study, Naylor et al. interviewed 1,129 members of the American Association of Endodontists regarding the use of bone graft substitutes. 62.9% of colleagues used bone graft substitutes to treat bone defects larger than 1 cm in diameter. 10.1% of colleagues used bone graft substitutes on defects smaller than 1 cm in diameter [19]. When considering the results of this study, however, it becomes clear that the use of bone replacement material to fill the defect after root tip resection probably has no influence on the success prognosis of the resected tooth. In this study, there was no uniform or clearly defined guideline for the use of bone replacement material for defect filling after root tip resection, which naturally reduces the expressiveness. Nevertheless, the results show quite clearly that the use of bone replacement material to fill the defect after root tip resection has no influence on the success prognosis of the resected tooth. This also corresponds to the results of Taschieri et al., in whose study the use of bone replacement material was compared with two control groups. They also found no significant difference between the success rates of both groups [20]. However, Dietrich et al. describe very good results over an observation period of twelve months in the therapy of apico-marginal defects using a bone substitute material (Bio Oss, Geistlich^®^) and a bioabsorbable membrane. But it should be noted that there was no control group in this study [21].

The therapeutic alternatives to root-tip resection are limited. The first therapy alternative is the revision of endodontic treatment. However, a revision of a root canal filling is usually very time-consuming and associated with high costs. However, a revision also brings advantages. For example, it avoids the need for a surgical procedure and prevents an additional loss of bone substance and thus the risk of tooth instability. In contrast, the revision of a root canal involves a certain risk of causing a “via falsa”. Instrument fractures and over-pressing of the root filling material can also occur. The literature contains many studies on the prognosis of endodontic revision treatments. They describe success rates of 62–89% [22], [23], [24]. There are also studies that compare the success rates of endodontic revisions with those of root tip resection. Both Kvist et al. and Torabinejad et al. describe an initially higher success rate for root tip resection. Over a longer observation period, however, a comparably high success prognosis for both procedures was found [25], [26].

With an increasing number of implantations performed over the years, extraction of the affected tooth with subsequent implantation should also considered as an alternative to root tip resection. Nowadays, the implantation of an artificial tooth root is becoming more and more important and is almost regarded as a routine procedure. However, this procedure involves a considerable financial expense for the patient. In addition to the financial costs, the time required cannot be neglected. While a tooth can be loaded directly after a root-tip resection and the patient ideally only has to make an appointment for a check-up or suture removal, an implantation involves several sessions and a waiting period of several months until the final implant-supported crown is completed. In contrast, the excellent 5- and 10-year long-term success rates for dental implants of over 90% support implant placement [27].

In addition to implantation, the conventional prosthetic replacement of the tooth after extraction, e.g. by means of a bridge, should of course also be mentioned. This also allows good functional and esthetic results to be achieved with a very good long-term prognosis [28].

## Conclusion

A root tip resection continues to be a good option for long-term tooth preservation after unsuccessful endodontic treatment. The success seems to be independent of the type of tooth. With increasing age, however, the long-term prognosis of root-tip resected teeth seems to decrease. The use of bone replacement material for defect filling can be dispensed with since this has no influence on the success of a root tip resection. Despite good 5- and 10-year success rates of root tip resected teeth, it is nevertheless advisable to strictly review the indication for root tip resection, since nowadays, for example dental implants offer a promising alternative.

## Notes

### Ethical statement

All procedures performed in this study involving human participants were in accordance with the ethical standards of the institutional and/or national research committee and with the 1964 Helsinki declaration and its later amendments or comparable ethical standards. No research/experimentation was performed on humans and/or animals for these case reports. The study was approved by the ethical committee of the University Ulm. Formal consent was not required for this type of study. 

### Competing interests

The authors declare that they have no competing interests.

## Figures and Tables

**Table 1:**
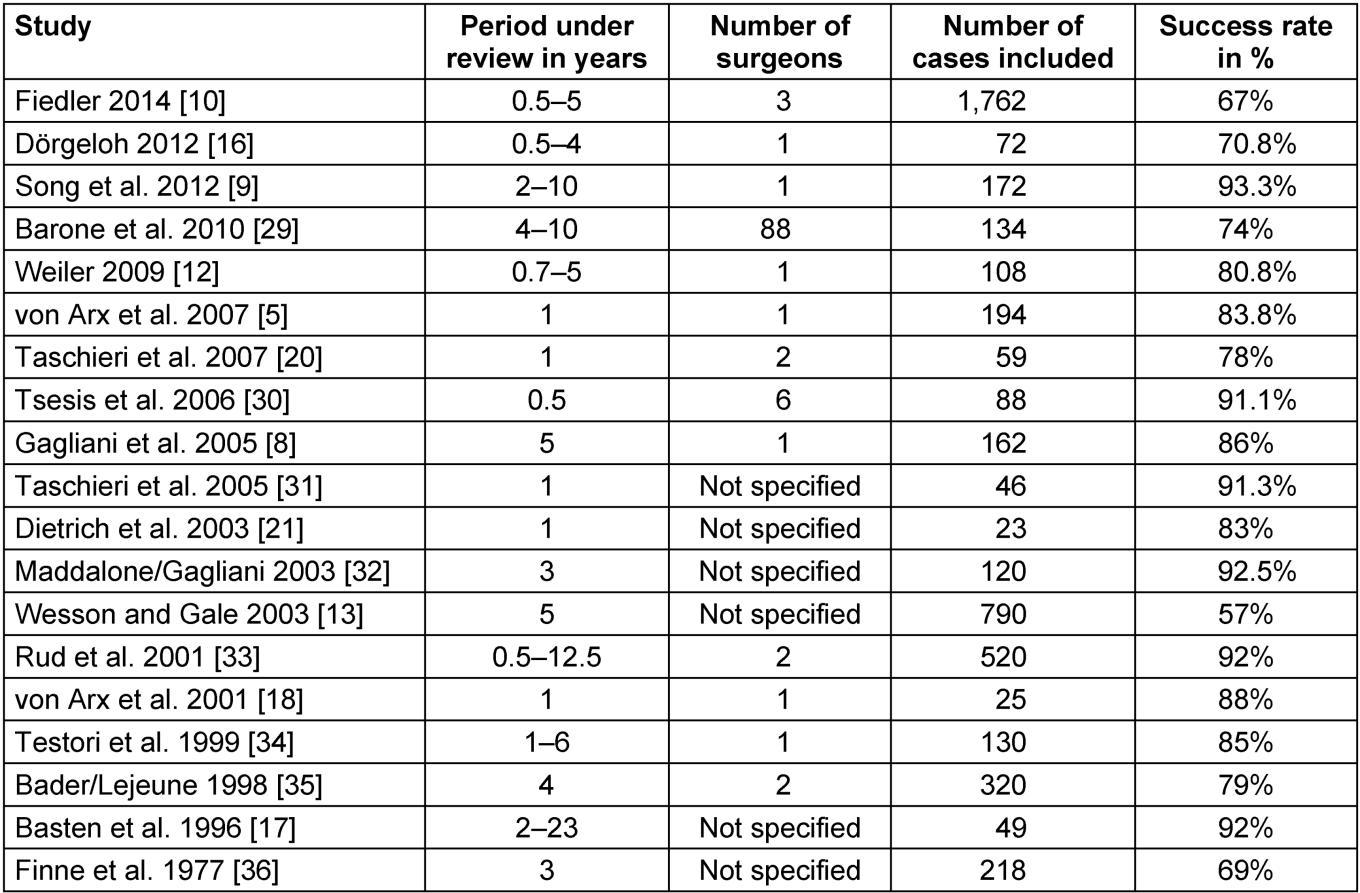
Presentation of comparative studies structured by period under review, number of surgeons, number of included cases and success rates
